# Sex-Specific Oral Health: A Narrative Review of Hormonal Influences and Disease Patterns

**DOI:** 10.3390/dj14030147

**Published:** 2026-03-06

**Authors:** Agnes Holtkamp, Florian Beuer, Thomas Gerhard Wolf, Michael Naumann

**Affiliations:** 1 Department of Prosthodontics, Geriatric Dentistry and Craniomandibular Disorders, Charité—Universitätsmedizin Berlin, 14197 Berlin, Germany; florian.beuer@charite.de (F.B.); michael.naumann@charite.de (M.N.); 2 Department of Restorative, Preventive and Pediatric Dentistry, School of Dental Medicine, University of Bern, 3010 Bern, Switzerland; thomas.wolf@unibe.ch; 3 Department of Periodontology and Operative Dentistry, University Medical Center of the Johannes Gutenberg-University Mainz, 55131 Mainz, Germany; 4 Private Practice, Zahnärzte an der Kleinmachnower Schleuse, Wannseestrasse 42, 14532 Stahnsdorf, Germany

**Keywords:** sex differences, oral health, hormones, estrogen, periodontitis, caries, menopause, gender-specific healthcare

## Abstract

This narrative review aims to analyze sex-specific differences in oral health, examine hormonal influences in women during puberty, pregnancy, and menopause, and compare oral health behaviors between men and women. Articles were selected based on: (1) sex-specific aspects of oral diseases, (2) hormonal influences on oral health, (3) comparative analyses between sexes, and (4) sex-disaggregated data on oral disease prevalence. Women experience hormonal vulnerabilities with estrogen deficiency causing xerostomia, mucosal atrophy, and increased caries susceptibility, showing parallels between oral and vaginal mucosa. Men demonstrate higher periodontitis prevalence (57% vs. 38%), utilize preventive services one-third less frequently, and show higher smoking rates (67% vs. 42%) and traumatic dental injuries (2:1 ratio). Women maintain better oral hygiene and treatment adherence. Sex-specific factors affecting both sexes remain unconsidered in dental practice. Women need targeted interventions during hormonal transitions, while men require improved preventive care engagement. Future research integrating sex-specific considerations is required to enable personalized oral health approaches for both sexes.

## 1. Introduction

Sex and gender are two different but closely interconnected concepts [1]. Sex encompasses three dimensions: the classification as male or female, the biological level (e.g., chromosomes, hormones), and the phenotypic sex, which includes external characteristics such as genitalia and body shapes [2]. Gender, on the other hand, refers to a social construct that describes identity, self-perception, and gender-related roles under the influence of cultural and social expectations [2,3]. Both sex and gender influence biological processes, immune response, and susceptibility to certain diseases [4]. However, there are additional factors that influence sex. For example, a deficiency of 5α-reductase (*SRD5A2*) is caused by mutations in the *SRD5A2* gene [5], with a prevalence of 1 in 4500 live births [6]. If *SRD5A2* is missing, testosterone cannot be converted to 5-dihydrotestosterone (DHT), which is responsible for the formation of internal and external genitalia [7]. A deficiency of 5α-reductase enzymes thus represents a molecular cause for deviations in sex development [8]. This is also referred to as a sexual differentiation disorder [9]. In individuals with XY chromosomes and this gene mutation, the testes are present at birth but remain inside the body, causing the external genitalia to resemble those of a female [10]. Only during puberty is enough testosterone produced for the external male sexual characteristics to develop [11]. This could explain why some people are not recognized in their sex.

For about ten years, the National Institutes of Health (NIH) has been calling for the inclusion of women and minority groups in clinical research. Since October 2014, applicants are even required to outline their plans for a balanced proportion of both sexes in their studies [12]. As a result, more than half of NIH study participants today are female, which has improved the understanding of sex-specific differences, at least within these studies [13]. However, clinical knowledge about diagnosis, treatment, and prevention of diseases continues to be based largely on studies conducted on male cells, animal models, and human subjects [14]. Despite suspected sex-specific differences, in vitro, preclinical (animal), and clinical studies are typically not analyzed in a sex-specific manner [15].

Data on differences in oral health between men and women come predominantly from cross-sectional studies, are often incomplete, and ambiguous. It is frequently unclear whether these differences are biological or socio-cultural in nature [16]. It is also unclear whether research findings are transferable between sexes. Appropriate statistical analysis—and of course, appropriate study design beforehand—should absolutely consider this aspect [17]. Thus, despite the establishment of the Office of Research on Women’s Health (ORWH) by the NIH in the U.S. over 20 years ago, sex-specific analyses in (pre-)clinical studies in medicine are often neglected and are, to the best of our knowledge, rarely discussed in dentistry. The focus on male subjects in animal studies [18] (such as, for example, animal studies in dogs in the field of implantology, osseointegration and periimplantitis) could obscure important differences between men and women in drug effects [19]. Men and women experience illness differently, which is reflected in both the incidence and prevalence of certain diseases. This phenomenon is also evident in the frequency and use of health resources [20].

Despite growing awareness of sex differences in medicine, oral health research and clinical practice continue to largely overlook the distinct biological and behavioral patterns between men and women. While hormonal influences on women’s oral health during puberty, pregnancy, and menopause are increasingly recognized, systematic integration of these factors into preventive and therapeutic strategies remains limited. Similarly, the higher disease burden and lower healthcare utilization among men warrant targeted attention. This integrative review aims to synthesize current evidence on sex-specific differences in oral health, focusing on hormonal influences throughout women’s lives and comparative disease patterns between sexes. By identifying knowledge gaps and existing evidence, we seek to inform the development of more personalized, sex-aware approaches to oral healthcare that address the specific needs of both women and men.

This review addresses three specific questions: (1) What is the specific impact of hormonal changes throughout women’s lives (puberty, pregnancy, menopause) on oral health outcomes? (2) How do sex-specific biological mechanisms (hormonal, immunological, genetic) and behavioral patterns contribute to differential oral disease prevalence between men and women? (3) What are the clinical implications for developing sex-aware preventive and therapeutic approaches in dental practice? By addressing these questions, we aim to synthesize current evidence and identify critical knowledge gaps that must be addressed to optimize oral healthcare for both sexes.

## 2. Material and Methods

The present integrative review combines studies with diverse methodologies to analyze sex-specific differences in oral health research. Literature search included all relevant publications up to February 2025. After defining the search terms, a comprehensive search was conducted electronically in PubMed, Embase and Cochrane library with additional hand search in gray literature in English language. This integrative review examines sex-specific differences in oral health patterns, focusing on the relationship between hormonal changes and oral health parameters in women at different stages of their life, with particular focus on menopause, including “urogenital syndrome” and analog suggesting an “orofacial syndrome.” The aim is to describe the state of sex-specific oral health research, draw attention to differences between the sexes, and identify possible therapeutic approaches.

The research questions were: (1) What is the specific impact of hormonal changes throughout women’s lives (puberty, pregnancy, menopause) on oral health outcomes? (2) How do sex-specific biological mechanisms (hormonal, immunological, genetic) and behavioral patterns contribute to differential oral disease prevalence between men and women? (3) What are the clinical implications for developing sex-aware preventive and therapeutic approaches in dental practice

Our literature search strategy was designed to accommodate the broad, interdisciplinary nature of this integrative review. Rather than using rigid predetermined search strings, we employed a flexible, iterative approach that allowed exploration across multiple thematic domains. These included hormonal influences on oral tissues and diseases (particularly estrogen effects during menopause, pregnancy, and puberty), sex-specific disease prevalence patterns (periodontitis, caries, xerostomia), and behavioral differences in oral healthcare utilization. The iterative methodology enabled us to follow emerging themes and citation trails, synthesizing diverse evidence types from cross-sectional studies, cohort analyses, and clinical observations relevant to understanding sex-specific considerations in oral health.

To enhance transparency while maintaining the flexibility inherent to integrative reviews, we note that our searches employed terms such as “sex differences oral health,” “estrogen periodontitis,” “menopause xerostomia,” “gender dental care,” “testosterone caries,” and “hormones salivary flow,” among others, adapted iteratively based on emerging findings. Given the narrative synthesis approach and the breadth of this interdisciplinary topic, we did not maintain formal tallies of records screened at each stage, as is typical for integrative reviews. The final synthesis incorporates findings from over 190 sources spanning epidemiological studies, clinical observations, and mechanistic research.

Inclusion and exclusion criteria: Studies were included if they: (1) reported sex-disaggregated data on oral health outcomes, (2) examined hormonal influences on oral tissues or diseases, (3) compared oral health behaviors or disease prevalence between sexes, or (4) investigated biological mechanisms underlying sex differences in oral health. We excluded studies that: (1) did not differentiate between male and female participants, (2) focused exclusively on pediatric populations (<12 years), or (3) were published in languages other than English. No restrictions were placed on publication date or study design to ensure comprehensive synthesis across this emerging research area.

Literature identification: In addition to database searches, we conducted manual screening of reference lists from key articles and relevant systematic reviews to identify additional sources. Gray literature searches included professional organization websites and conference proceedings in oral health and women’s health.

Given the narrative synthesis approach of integrative reviews and the diversity of included study designs (cross-sectional, cohort, case–control, clinical observations), we did not employ formal quality appraisal tools or double extraction procedures typical of systematic reviews. Instead, data synthesis focused on identifying patterns, themes, and convergent findings across the heterogeneous literature base.

Articles were thematically organized according to life stage (puberty, pregnancy, menopause), disease category (caries, periodontitis), biological mechanisms (hormonal, immunological), and behavioral factors. Data extraction focused on sex-specific prevalence rates, hormonal effects, biological mechanisms, and behavioral patterns relevant to the review questions.

## 3. Findings from the Literature

### 3.1. Hormonal Changes and Oral Health: Three Phases in a Woman’s Life

Throughout their lives, women undergo three significant hormonal changes with puberty, pregnancy, and menopause, which strongly influence their psychological and physical well-being [21,22,23,24]. Biomedical research has identified significant sex differences in health patterns and has investigated, among other things, the effects of pregnancy on oral health [25] and associated quality of life [26]. During pregnancy, oral diseases such as gingivitis, periodontitis associated with tooth mobility, erosions, and caries temporarily occur frequently [27]. Menopausal changes have a lasting impact on a woman’s oral health and therefore require special attention [28]. Menopause is defined as the absence of menstruation due to the cessation of follicular maturation in the ovaries and is clinically diagnosed after 12 months of continuous amenorrhea [29]. In western countries women reach menopause, their last menstrual period, at an average age of about 51 years. Female smokers, diabetics, and women with many children tend to reach it somewhat earlier [30]. This entire period of adjustment and restructuring lasts 10 to 15 years, is called in medical context as climacterium, which translated from Greek means “ladder”. Menopause may be distinguished as follows [30]:

1. Premenopause (duration approx. 4 years): The hormone balance changes, but menstruation still occurs regularly.
2. Perimenopause (duration approx. 6–7 years): During this “peak phase,” the ovaries cease their activity. Irregularities in the cycle occur, up to the absence of menstruation (=menopause).
3. Postmenopause (begins 12 months after the last period): After menopause, hormone levels stabilize at a new level. Depending on the onset of the last menstruation, it is classified as premature (before age of 40), early (between age 40 and 45), or late menopause (after age of 55).

A cohort study with 914 women aged 40 to 65 years found a relationship between different dietary patterns and the age at onset of menopause [30]. While a diet with a high glycemic index was associated with menopause occurring on average 1.5 years earlier, a good supply of vitamin B6, vitamin D or zinc, and a diet rich in fatty fish and legumes was associated with a menopause that occurred a few months later. There appears to be a connection between the timing of menopause and the occurrence of osteoporosis, coronary heart disease, and depression [31]. The earlier the last period, the higher the risk for each of these diseases, and overall mortality increases. A later onset of menopause, on the other hand, exposes the female body to estrogens for a longer period, which may increase the risk of hormone-related cancers, particularly breast, uterine, and ovarian cancer [32].

In addition to systemic diseases such as coronary heart disease, osteoporosis, arthritis, cognitive decline, and Alzheimer’s, estrogen deficiency after menopause can also lead to oral changes such as xerostomia, burning mouth, increased susceptibility to caries, dysesthesia, taste disorders, atrophic gingivitis, and periodontitis [33]. About 85% of menopausal women experience hot flashes. The hormonal changes after the onset of menopause are viewed as “natural” and to be accepted. They are usually reduced or “trivialized” to mood swings and hot flashes. This perspective, at least in Western society, likely massively underestimates the sometimes severe individual psychological suffering, health limitations (“urogenital syndrome”: dry vagina, urinary incontinence, and frequent bladder infections), and resulting health economic costs (workplace productivity, doctor visits, disease costs, medication costs for substitution and therapies) [34,35,36], especially considering the fact that women today have a similar life expectancy before and after menopause.

Based on just described relationships, it appears equally justified to use the term “orofacial syndrome” comparable to the “urogenital syndrome,” since dry mouth, burning mouth, caries and periodontitis tendency, as well as facial pain and temporomandibular joint disorders may occur more frequent in peri- and postmenopausal women [4,37,38,39]. It seems urgently necessary that dental studies in future and retrospectively will be analyzed in a sex-specific manner. The long-term task is to improve prevention, diagnosis, and treatment of estrogen deficiency-associated complaints and diseases. The focus is on the hormonal factors that a woman is exposed to throughout her life.

### 3.2. Sex-Specific Differences in Oral Healthcare Behaviors

Studies show that men demonstrate significantly lower healthcare utilization across multiple domains, accessing fewer medical appointments, emergency services, preventive care visits and hospice services, while also experiencing shorter hospital stays [40,41]. Men also visit dentists less frequently and more often neglect their oral health [42]. This reduced healthcare engagement contributes to decreased life expectancy among the male population. According to the National Ambulatory Medical Care Survey of 2007, men use preventive services about one-third less frequently than women. Additionally, almost 60% of men avoid treatment even if they are at risk of suffering from a serious illness [43]. They usually seek dental help only for acute problems, while women tend to adhere to recommended treatments after an examination [44]. Although women more often feel anxious about dental visits [45], they nevertheless show an overall more positive attitude toward dental visits [46], a higher health literacy, and better oral hygiene than men. This leads, among other things, to more frequent tooth brushing and better care [44,47]. Habitual differences, such as smoking behavior, play a crucial role in oral health [48]. A study with Thai university students shows that men are more likely to smoke daily compared to women (67% vs. 42%) [49]. Cigarette consumption is associated with an increased risk of oral and pharyngeal cancer as well as gingival and periodontal diseases [50]. Studies suggest that smoking alters the oral microbiome, thereby increasing the risk of caries [51,52] and leading to reduced saliva production and altered saliva composition [53]. Smoking is also suspected to be a significant risk factor for the loss of periodontal attachment apparatus and tooth loss [50,54]. Another difference between the sexes is the increased occurrence of traumatic dental injuries in boys compared to girls (ratio of 2:1), which is attributed to behavioral factors: boys participate more often in outdoor activities, contact sports, and are more frequently involved in violent confrontations [55].

### 3.3. Sex Differences in Common Oral Diseases

#### 3.3.1. Caries

Worldwide, caries is one of the most common chronic diseases of the oral cavity and is considered one of the most widespread diseases in general [56]. The reasons for this are often multifaceted and multifactorial [57]. It is assumed that caries arises from the interaction between bacterial biofilm and acid-forming carbohydrates [58]. Risk factors include high bacterial load, poor oral hygiene [59], inadequate fluoride intake [60], and poverty [61]. Saliva, sex-specific genetic predispositions, and hormonal influences also play a role [44]. Saliva protects the teeth from caries and the mucous membranes, supports chewing, swallowing, speech, and promotes the maintenance of the natural microbiome. It is essential for oral and general health [62]. Diseases, hormones, and medications can impair saliva secretion, leading to xerostomia, caries, and fungal infections [62,63]. Caries is seen as the main cause of tooth loss [64,65]. The prevalence of caries varies between the sexes. Compared to men, women are more frequently affected by caries [66,67]. A study based on a Swedish population group demonstrates that even in the early modern period, women had a significantly higher caries experience than men [68]. It is assumed that certain predispositions to caries development in women are encoded on the X chromosome [4]. Since about 90% of amelogenin is expressed from the X chromosome, a deficiency can weaken the tooth enamel surface and increase susceptibility to caries [69]. Another discussed reason is the earlier tooth eruption in girls, which exposes the teeth longer to a potentially caries-promoting oral environment [44]. In contrast to women, men are more strongly affected by root caries [70]. This is attributed to a more aggressive brushing technique and poor oral hygiene, which promotes gingival recession and thus secondarily the development of root caries [71]. Furthermore, the omission of daily fluoride application, as is often the case with men, leads to increased susceptibility to cariogenic processes [71].

Diabetes is also associated with the development of carious lesions [72], making this systemic disease locally significant for oral health. In diabetes, typically referring to type 2 diabetes (T2D), high blood sugar levels can increase the amount of glucose in saliva [73]. This promotes the growth of bacteria and the formation of plaque, which favors the development of caries [74]. Diabetes can also lead to reduced saliva production (xerostomia) [75], another factor that secondarily contributes to the development of caries, as saliva neutralizes acids and cleanses the oral cavity [76]. A lower amount of saliva consequently increases the risk of caries [73]. Biological sex differences in the clinical course of the disease in patients with type 2 diabetes (T2D) are attributed to genetic disposition, hormonal influences, diagnosis, and response to therapeutic measures [77]. Men, especially in younger years, have a slightly increased overall risk of developing T2D [78]. One reason for the higher prevalence in men is probably the larger proportion of trunk and visceral fat, more fat mass in the upper extremities, and a higher liver fat concentration compared to women of the same age [79]. Testosterone deficiency is another factor that promotes the development of T2D in men, while an excess of androgens increases the risk of type 2 diabetes in women [80].

Functional hypogonadism can occur because of obesity. Serum testosterone levels below 16 nmol/L increase the risk of men developing type 2 diabetes (T2D). Higher testosterone levels, on the other hand, show a protective effect. Interestingly, weight reduction is directly related to an increase in serum testosterone concentration and thus increases the chance of preventing T2D or reversing a newly diagnosed disease [81]. Men and women with similar degrees of insulin resistance show similar amounts of intra-abdominal fat and liver fat [82]. Women with T2D are at increased risk for cardiovascular and metabolic diseases [83] compared to women without T2D. In contrast to women, a pooled analysis of prospective studies found that men with T2D have a significantly higher risk of cancer-related mortality than men without T2D [84]. The T2D-related sequelae vary between the sexes and are influenced by sex-specific factors such as psychosocial and cultural differences, behaviors, lifestyles, and attitudes, prevention, susceptibility, and disease progression [77]. Female obesity is more common in countries with pronounced gender inequality [85]. In industrialized countries, income inequality correlates with obesity and diabetes mortality rates in both sexes, with women being more affected than men [86]. It is notable that women are more likely to be overweight or obese only from the age of 45, while men tend to have higher overweight already at younger ages [87]. In women, the risk increases during certain life phases, such as after menopause or during pregnancy [88]. Obesity is the main risk factor for T2D in both sexes, which is why the prevalence patterns are regionally proportional to obesity. The globally standardized diabetes prevalence in adults aged 20 to 79 years (as of UN world population 2021) is estimated at 10.5% (SD 8.3–12.0%) (m 10.8%; f 10.2%) [89]. From the relationships described above, an epidemiologically relevant risk for carious diseases and simple methods for prevention (e.g., preventing obesity) and therapy (e.g., weight reduction) can be derived.

Menopause is another factor that can increase the risk of caries [90]. Hormonal changes, particularly the decline in estrogen and progesterone [91], influence various aspects of oral health. A decreasing estrogen level is often associated with reduced saliva production (xerostomia) [92,93], which, as an additional factor alongside other risk factors such as T2D, contributes to the development of caries. In addition to mucosal atrophy in menopause [90], gingival recessions also occur more frequently, and they are observed significantly more often in women with osteoporosis than in women without osteoporosis [94]. This, in turn, exposes the surface of the tooth root, or root cementum, at least partially, which is less resistant to cariogenic attacks than tooth enamel. This increases the risk of root caries [95]. Women show an increased risk of depression during or after menopause [96]. Since it is known that emotional components can also impair oral hygiene behavior in different life phases, this promotes the development of caries [97].

The risk of caries can also be increased during puberty. In addition to dietary habits such as high sugar consumption and inadequate oral hygiene during this life phase [98], the estrogen level of both sexes increases during puberty, with the increase being much more pronounced in women and playing a central role in the development of secondary sexual characteristics [99]. Hormonal changes during puberty have significant effects on oral health and increase the risk of caries [100]. The increased blood flow and vascular permeability of the gingiva because of hormonal fluctuations favor the development of gingivitis, although adequate brushing habits can compensate for this [101]. The estrogen level fluctuates in women during different life stages such as puberty, pregnancy, and menopause, which can increase the risk of depressive moods, anxiety, and physical complaints such as fatigue and pain [102,103]. Similarly to menopause, women develop a sex-specific difference in depression risk compared to men during the pubertal transition, with girls being about twice as likely to be affected by depressive episodes as boys [104,105]; another risk factor that can promote the development of caries [106].

Pregnant women are exposed to an increased risk of caries in many respects [107]. In the case of pregnancy-related nausea (hyperemesis gravidarum), acidic reflux leads to tooth enamel erosion due to the effect of stomach acid [108]. Due to the increased acid content in the mouth, cravings for sweets, and possibly reduced attention to oral hygiene, they are particularly at risk [109]. Not only pregnant women but also their children, especially with a high caries burden of the mother, have an increased risk of developing caries themselves [110] (Table 1).

#### 3.3.2. Periodontitis

Gingivitis is the most common oral disease during pregnancy, with a prevalence of 30 to 100% [112,113]. Women who already suffered from gingivitis before pregnancy experience a significant worsening during pregnancy [114]. The etiology of periodontal diseases is complex and not yet fully understood [115]. Gingivitis and periodontitis are widespread among adults, show age-related progression, and are supposed to be related to systemic diseases [116,117]. Diseases of the periodontium are pathological processes, usually developing chronically and progressively. One reason for this is probably, among other things, the accumulation of bacterial biofilms (plaque), which induce microbial dysbiosis [118] and thus favor the formation of periodontal pockets. Resulting gingival recessions, the progressive degeneration of the periodontal tissue, and the resorption of the alveolar bone can ultimately lead to tooth loss [119]. The frequency of periodontitis is significantly higher in men at about 57% compared to women at about 38% [111] and shows a significantly higher incidence of severe periodontitis across all age groups and educational levels [120]. The inflammatory reactions in periodontitis suggest that differences in the immune response influence the course of the disease, with evidence suggesting that men tend to show a less robust immune reaction to pathogenic microorganisms [121,122]. This could explain the sex-specific divergences in the immune reaction to oral pathogens and, at least partially, the lower prevalence of periodontal diseases in women [123]. Sex hormones and X-chromosomal genes also likely play a central role in the immune response [117]. The sex chromosomes influence the immune reaction, with X-chromosomal genes in particular playing a modulating role. In addition, sex hormones such as estrogen, progesterone, and testosterone have effects on immunity [44]. The effect of sex steroids on immune function depends both on the concentration of the respective hormone and on the cellular expression of the corresponding hormone receptor [124]. Thus, the estrogen concentration can either enhance or suppress the immune response depending on the ligand, receptor, and cell type [117]. Periodontal diseases can negatively influence the course of a pregnancy [125]. The pathogenic subgingival microorganisms favor translocation into the bloodstream, causing bacteremia [126]. The increased inflammatory mediator production due to periodontitis affects the placental barrier, allowing the transfer of oral microorganisms and toxic components. This interaction favors the infection of the fetoplacental unit [126]. Some risk factors, such as T2D and obesity, which generally occur more frequently in men, support the male predisposition for periodontal diseases [4]. T2D and periodontitis are interconnected, widespread worldwide, and significantly affect the quality of life of affected individuals [127]. In addition to periodontitis, there are other consequences of poorly controlled blood sugar levels. Inadequate blood sugar control in diabetics, measured by the value of glycated hemoglobin (HbA1c), is closely related to micro- and macrovascular complications [128]. T2D is characterized by a disturbed, hyperglycemic metabolic state and is associated with a shortened life expectancy. The most common complications include cardiovascular diseases [128], diabetic retinopathy, and kidney failure [129]. Studies show that T2D increases the risk of periodontitis in adults. The bidirectional relationship between both diseases is well documented [130,131]. T2D can promote periodontitis, while periodontitis can worsen blood sugar levels in diabetics [131,132]. Due to the increased tendency to inflammation in diabetes, the gingiva and the supporting tissue of the teeth are attacked, which in turn favors periodontitis [133]. Other causes for gender-specific differences could be superior oral hygiene, differing behavioral risk factors such as nicotine consumption [50] and stress, more frequent dental consultations, and higher compliance with therapeutic measures among women [134]. Interestingly, a sex-specific data analysis shows that severe periodontitis occurs more frequently in women from the age of 65 [120]. However, there are also contradictory study results, possibly due to different disease definitions, examination procedures, or selection biases of the sample population [4].

It is discussed that sex steroid hormones could play a decisive role in the development of periodontal diseases [135], as they modulate the reactions of the periodontal tissue and its response to the microbial biofilm, contributing to the pathogenesis. These hormones influence the periodontal attachment apparatus in life phases such as puberty, pregnancy [136], menstruation, and menopause [135,137]. Estrogen has versatile effects depending on the concentration. In women, an elevated estrogen level favors the development of gingivitis [138]. A low estrogen level leads to the loss of alveolar bone [139] and contributes to mucosal atrophy [140]. During pregnancy, bidirectional and reciprocal effects of periodontitis are observed. Women are more susceptible to inflammation during pregnancy, which often begins in the second or third month of pregnancy. The gums are often reddened, swollen, sensitive, enlarged, and tend to bleed when touched [141]. Adverse pregnancy outcomes are a relevant health problem with social and economic implications [142]. Studies suggest a possible connection with periodontal diseases in pregnant women. Premature births, defined as births before the 37th week of pregnancy (<259 days), are considered the most common cause of neonatal deaths in the first four weeks of life [143]. A birth weight of less than 2500 g (low birth weight) is often observed in premature babies or children with intrauterine growth disorders [144]. Periodontitis increases the risk of premature births and low birth weight, as chronic inflammation and the release of proinflammatory cytokines such as IL-6 and TNF-α can trigger premature labor [145]. But also, the hormonal changes during pregnancy itself and the increase in progesterone and estrogen can influence cytokine production. This elevated hormone level promotes the release of proinflammatory cytokines such as IL-6, IL-8, and IL-1β, which can influence the prevalence and severity of gingival inflammation [142]. Progesterone increases the synthesis of prostaglandins, especially Prostaglandin E2 (PGE2), which can intensify the symptoms of gingival inflammation through increased capillary density and vascular permeability [146]. Pregnant women with periodontitis have elevated IL-6 and TNF-α levels in saliva and blood compared to healthy individuals [147]. Bacteria and proinflammatory cytokines from infected periodontal tissue enter the systemic circulation and can increase the C-reactive protein level through an acute liver reaction [148]. Elevated plasma levels of C-reactive protein in pregnant women enhance an intrauterine inflammatory reaction, promote tissue damage, and the release of proinflammatory cytokines. These increase uterine contractility, cause premature cervical ripening, endothelial dysfunction, and membrane ruptures. This increases the risk of intrauterine restricted growth conditions for the unborn child, low birth weight, premature births, and preeclampsia (PE) [142,149]. PE is a complication in pregnancy characterized by newly occurring high blood pressure, often with proteinuria, and usually occurs after the 20th week of pregnancy or shortly before birth [150]. Pre-eclampsia (PE) is considered a common multisystemic pregnancy disorder and occurs in 2–8% of pregnancies worldwide [151]. It is responsible for about 50,000 deaths in pregnant women and 500,000 deaths in fetuses and newborns each year, thus representing an epidemiologically relevant, very serious risk to the health of mother and child [152]. The systemic inflammatory reaction favors the risk of PE [153], possibly through multiple bacterial loads in the subgingival plaque in periodontitis, which interact with the epithelium of the periodontal pockets and reach the fetoplacental unit via the bloodstream [126,154]. The pathogenesis is not yet fully understood [155]. However, it is assumed that it is influenced by a combination of individual health status, immunological reactions, and environmental influences during pregnancy [156]. Studies on the connection between periodontitis and PE provide contradictory results. Some study results show that periodontitis and PE are not related [125]. Others point to an increased PE risk due to poor periodontal health [157,158] and present periodontitis as a significant risk factor, especially in low- and middle-income countries [153] (Table 2).

### 3.4. Estrogen with Similar Significance for Oral and Vaginal Mucosa

To the best of our knowledge, the aspect of menopause and the postmenopausal period as an influencing factor on intraoral symptoms is scientifically underrepresented. According to the WHO, menopause is the last menstrual bleeding, which usually occurs between 45 and 56 years and then remains absent for 12 months. Since women have a higher life expectancy than men, they spend an average of about 40% of their lives, most women even over 30 years, after menopause [168]. Hormonal changes during menopause affect the body in the same way as hormonal changes in pregnancy. Estrogen influences the oral mucosa directly or through neuronal mechanisms and thus affects periodontal health in women during menopause [169]. Postmenopausal women are more frequently and severely affected by periodontal diseases [159]. Xerostomia or dry mouth often occurs in peri- and postmenopause [160]. The decline in serum estrogen levels plays a central role in this, as both the oral mucosa and the salivary glands contain estrogen receptors [162]. Steroid hormones in saliva correlate with those in blood serum. Thus, the salivary flow rate depends on the estrogen status [162,163]. The subtype of the estrogen receptor (eRβ) in the oral epithelium is responsible for the maturation of the epithelium [162]. In women with dry mouth during menopause, the average estrogen level is significantly lower [170]. Many women report xerostomia, a subjective feeling of decreased salivary flow, taste disorder, burning mouth, a changed pain perception, and a reduced acceptance of removable prosthetic restorations [90,171]. Xerostomia occurs mainly in women in peri- and postmenopause, especially in those over 50 years old. Asplund & Aberg reported that 17.8% of women between 40 and 64 years complained about dryness before menopause, 23.3% during and up to 5 years afterward. Five to 9 years after menopause, it was 29.2%, and over 10 years later, 34.5% [161]. The decreased salivary flow is associated with menopause but also often occurs with age. Additionally, systemic diseases such as diabetes, rheumatoid arthritis, Sjögren’s syndrome, as well as the regular intake of medications that have xerostomia as a side effect, intensify the feeling of dry mouth in women during menopause [63]. The decline of estrogens in the salivary glands leads not only to quantitative but also to qualitative changes [172] and makes the oral mucosa more susceptible to mechanical stress such as abrasions, tears, infections, and candidiasis [161,173]. Due to the decreased estrogen level, the calcium concentration in saliva also increases [164,165]. This favors plaque mineralization and tartar formation, which in turn increases the risk of gingivitis and periodontitis [166]. Premenopausal women have a higher salivary flow and an increased concentration of immunoglobulins such as IgM and IgA compared to postmenopausal women [63]. In contradiction to this, another study could not prove a significant decrease in salivary flow in women during menopause [174].

Both in the vaginal mucosa [175] and in the oral mucosa, estrogen deficiency during menopause leads to a thinning and atrophy of the epithelium, which increases susceptibility to inflammatory processes [63,162]. It is assumed that estrogen receptors in the oral mucosa and salivary glands respond to hormonal influences. The similar histology between oral and vaginal mucosa explains the common cause of the symptoms [63]. Analogous to the oral cavity, menopausal hypo-estrogenism also significantly affects the health of the vagina and urinary tract and often leads to genitourinary syndrome (GSM) [176]. The GSM of menopause is characterized by symptoms such as dryness, burning, irritation, as well as pain or discomfort during sexual intercourse, and impaired sexual function; also called vulvovaginal atrophy (VVA) [177]. The falling estrogen level during menopause is particularly considered as the main trigger of VVA [178]. The most common symptoms include vaginal dryness (78%), which is associated with significantly reduced sexual activity [175], superficial dyspareunia (76%), burning, pain, and itching, especially in the vulvar area [179]. There are also parallels to the oral mucosa, such as burning mouth syndrome (BMS). It predominantly affects women in perimenopause or after menopause and causes burning pain in the oral mucosa that lasts for several months. In addition to the physical change, a connection with mental disorders and nervous system changes [180], sleep disorders, irritability, depression, and anxiety states are also discussed as reasons for the pain in the oral mucosa [181,182]. A significant correlation was found between the intensity of psychosomatic symptoms and a low socio-economic status of women, independent of age, ethnicity, menopause, and possible hormone replacement therapy [183] (Table 3).

### 3.5. Estrogen and Implant Therapy

Another phenomenon that correlates with the decline in estrogen levels is the reduction in bone density [167]. Particularly in connection with the probability of success of dental implants, there is little good evidence regarding osteoporosis or bone density in the context of estrogen, estrogen deficiency, and the benefit of hormone replacement therapy. Thus, the healing of the alveolar bone in the context of implantation is said not to be influenced by temporary changes in the ovarian cycle [184]. A correlation between peri-implant bone loss and hormone replacement therapy (HRT, hormone replacement therapy) (*p* = 0.002) could be shown. Therefore, patients who want to replace missing teeth with implants and receive hormone replacement therapy should be informed about an increased risk of osseointegration failure and thus the success of implant therapy [185]. It could be shown that the postmenopausal estrogen status has a negative influence on implant healing in the upper jaw but not in the lower jaw. Non-supplemented postmenopausal women have the highest implant failure rate. Although not statistically significant, HRT reduced the failure rate in the upper jaw by 41%. It was concluded that an estrogen deficiency and resulting bone changes associated with menopause could be a risk factor for implant failures in the upper jaw [186]. In contrast, another study showed that HRT is not associated with an improved outcome of implant therapy in postmenopausal women [187]. Systemically applied medications can fundamentally have an improving or deteriorating influence on the osseointegration of dental implants, although these processes are not yet fully understood [188]. The situation is similar, for example, with osteoporosis and osseointegration. A systematic review reports on 17 publications of osteoporotic patients with heterogeneous results. However, the evidence for an association between osteoporosis and implant failure was low [189]. Prospective controlled studies are urgently needed, especially for patients with manifest osteoporosis undergoing bisphosphonate therapy [190]. A systematic review and meta-analysis conclude that implants are an acceptable treatment option for patients with osteoporosis [191]. Another work of this kind found no difference between patients with and without osteoporosis regarding the survival probability of implants, neither at the implant level (RR 1.39, 95% CI 0.93–2.08; *p* = 0.11), nor at the patient level (RR 0.98, 95% CI 0.50–1.89; *p* = 0.94). However, the meta-analysis showed a significant difference regarding marginal bone loss around implants in patients with and without osteoporosis (0.18 mm, 95% CI 0.05–0.30, *p* = 0.005) [192].

### 3.6. Estrogen and Aspects of Nutrition

Until the age of 35, bone formation predominates over bone resorption, with estrogen acting as a protective factor. After the age of 35, bone resorption tends to predominate due to decreasing estrogen concentration and is strongest in the five years before and after menopause. To slow down the degradation process, calcium and vitamin D are essential [193,194]. In addition, plenty of exercise and the reduction in sugar intake and highly processed products are recommended. This has positive effects on bone mineralization and mass. Furthermore, there is evidence that a Mediterranean diet, thanks to its content of beta-carotene, vitamin C, selenium, and other minerals and vitamins, has a protective effect on the skeleton and supports bone mineralization [194]. Observational studies in countries with high consumption of soy products also show lower prevalence of osteoporosis among postmenopausal women than in those where soy hardly plays a role in nutrition. The phytoestrogens contained in soy, particularly isoflavones, are often discussed as an explanation. The latter resemble estrogen in their chemical structure. The extent to which isoflavones in soy products as part of the diet or in the form of dietary supplements are actually associated with hard endpoints such as lower fracture rates in women during and after menopause still needs to be investigated [195].

## 4. Discussion

This integrative review reveals significant sex-specific patterns in oral health requiring comprehensive understanding of both biological and behavioral factors. The analysis demonstrates that hormonal fluctuations throughout women’s lives significantly impact oral health, while men exhibit distinct patterns of disease prevalence and healthcare utilization [20]

Women’s three major hormonal transitions—puberty, pregnancy, and menopause [21,22,23]—create unique vulnerabilities. The concept of an “orofacial syndrome” paralleling the urogenital syndrome of menopause [176] provides a framework for understanding systemic effects of estrogen deficiency on oral tissues [4,37,38,39]. Women demonstrate higher caries prevalence [66,67] but maintain better oral hygiene practices and seek preventive care more frequently [44,46,47].

Men show significantly higher periodontitis rates (57% vs. 38%) [111] with concerning preventive care avoidance patterns [40,41,42]. Approximately 60% avoid treatment even when at risk of serious illness [43], utilizing preventive services about one-third less frequently than women. Behavioral factors including higher smoking rates (67% vs. 42%) [49] and increased traumatic dental injuries (2:1 ratio) [55] contribute to distinct challenges.

Sex hormones and X-chromosomal genes play central roles in immune responses and oral health outcomes [44,117,124]. Differential immune responses, with men showing less robust reactions to pathogenic microorganisms [121,122], partially explain higher severe periodontal disease prevalence [123]. Estrogen’s multifaceted effects on salivary flow [162,163], epithelial maturation [162], and immune function [117] create sex-specific vulnerabilities and protective factors.

### 4.1. Limitations

There are several limitations that affect this review. Study design and population heterogeneity limits direct comparisons [16]. Many studies lack adequate sex-specific analyses [15], and behavioral factors often confound biological differences. The predominant focus on women in hormonal research creates gaps in understanding male-specific patterns [18]. Cross-sectional designs limit causal inferences [17]. Additionally, we did not employ formal quality appraisal tools, and database coverage was limited to English-language publications, introducing potential publication bias.

### 4.2. Implications of Clinical Practice and Future Research

Thus, various necessities arise for clinical practice:

Women in menopause require intensified care oriented toward prevention and treatment of oral diseases [28], including regular check-ups and education about xerostomia [160,170], mucosal atrophy [90,140], and periodontal diseases [159]. The psychosocial dimension, particularly depression and stress during menopause [96,181,182], should be integrated into counseling, as these factors influence oral hygiene behavior and disease development [97].

For men, prevention strategies should improve engagement with preventive services [40,41,42] and address behavioral risk factors through targeted smoking cessation programs [48,50], periodontal disease education [111,120], and male-friendly approaches acknowledging their tendency to seek care only for acute problems [44]. Recent findings point to gender-specific correlations between caries, tooth loss, and overweight/obesity, with stronger and more pronounced BMI-related gradients observed in women [196]. This underscores the need for gender-specific and gender-equitable prevention strategies that take common risk factors into account [196].

Gender-specific prevention strategies must consider both biological and social determinants [16,17]. Given hormonal effects on women’s oral health [21,22,23,24], research should urgently differentiate sex-specific data and results [15]. Only through stronger integration of both sexes in clinical studies [12,13] and precise investigation of their needs across life phases can healthcare be optimized.

Interdisciplinary collaboration between dentists, internists, gynecologists, and psychologists appears particularly promising to ensure comprehensive care tailored to the needs of both women and men. Apart from biological considerations, sex and gender are important aspects of patient-centered care. It is really important to integrate the basic principles of sex- and gender-sensitive healthcare into dental education and clinical practice in order to promote inclusive dental care and respond appropriately to the needs of patients with different gender identities and gender expressions [197].

## 5. Conclusions

The study of sex-specific differences in oral health, particularly during puberty, pregnancy, and the phases before, during, and after menopause, reveals a significant gap in the focus on comprehensive oral health research that adequately addresses both sexes. Hormonal changes during these life stages, especially fluctuations in estrogen levels, have a profound impact on women’s oral health. These changes significantly influence the prevalence and severity of conditions such as caries, gingivitis, periodontitis, xerostomia and its complications and increase the risk of bone density loss and the associated medications, which have clinical implications, e.g., for implant therapy. Future research must focus on a better understanding of the biological mechanisms that affect oral health throughout key life stages for both sexes. The integration of sex-specific considerations into oral healthcare represents an essential step toward personalized medicine approaches that acknowledge the biological and behavioral differences between men and women while ensuring optimal oral health outcomes for all patients.

## Figures and Tables

**Table 1:** Sex-Specific Differences in Oral Disease Prevalence and Risk Factors.

Parameter	Men	Women	References
Periodontitis prevalence	57%	38%	[111]
Caries prevalence	Lower	Higher	[66,67]
Root caries	More affected	Less affected	[70]
Smoking rates	67%	42%	[49]
Traumatic dental injuries	2:1 ratio (boys:girls)	-	[55]
Preventive service utilization	33% less than women	Baseline	[43]
Treatment avoidance (despite risk)	~60%	Lower	[43]
Oral hygiene practices	Poorer	Better	[44,47]

**Table 2:** Hormonal Influences on Women’s Oral Health Across Life Stages.

Life Stage	Hormonal Changes	Oral Health Effects	References
Puberty	↑ Estrogen	Increased gingivitis risk; enhanced gingival blood flow and vascular permeability; elevated depression risk (2× vs. boys)	[99,100,101,104,105]
Pregnancy	↑ Estrogen, ↑ Progesterone	Gingivitis (30–100% prevalence); increased caries risk; risk of premature birth and low birth weight; preeclampsia risk	[112,113,114,142,145,153]
Menopause	↓ Estrogen, ↓ Progesterone	Xerostomia (17.8–34.5% depending on time since menopause); mucosal atrophy; increased caries and periodontitis risk; gingival recession; burning mouth syndrome	[90,92,93,140,159,160,161]
Postmenopause	Sustained ↓ Estrogen	Reduced salivary flow; increased calcium in saliva; enhanced plaque mineralization; bone density reduction	[162,163,164,165,166,167]

**Table 3:** Summary of Key Biological and Behavioral Mechanisms.

Factor	Sex-Specific Pattern	Clinical Relevance	References
Immune Response	Men: Less robust immune reaction to pathogens Women: Stronger immune response	Contributes to higher periodontitis in men; X-chromosomal genes modulate immunity	[117,121,122,123]
Estrogen Receptors	Present in oral mucosa and salivary glands (ERβ subtype predominant)	Influences epithelial maturation, salivary flow, and tissue response	[162]
Healthcare Behavior	Men: Reactive care-seeking Women: Proactive preventive care	Men visit dentists less frequently; Women show better treatment adherence	[40,41,42,44,46]
Risk Behaviors	Men: Higher smoking rates, more traumatic injuries Women: Better oral hygiene	Compounds biological susceptibilities	[48,49,50,55]
Diabetes & Oral Health	Men: Slightly higher T2D risk at younger ages Women: Higher CVD risk with T2D	Bidirectional relationship between periodontitis and T2D affects both sexes	[78,83,127,130,131,132]

## Data Availability

No new data were created or analyzed in this study. Data sharing is not applicable to this article.
